# Supplementary material for: Potential contribution of fish restocking to the recovery of deteriorated coral reefs: an alternative restoration method?
Source: PeerJ. 2016 Feb 29;4:e1732. doi: 10.7717/peerj.1732 (PMC4782690; doi:10.7717/peerj.1732)
Supplement: Text S1 [file peerj-04-1732-s001.docx]

**Spillover estimates**

([Kaunda-Arara and Rose 2004](#_ENREF_1)) observed a 6.3 and a 10 km^2^ coral reefs (with a distance of ~25 km between them). They estimated the spillover by seeing how much of tagged fish were trapped inside and outside a park (weighted by the number of fish traps in each location). Spillover=$\frac{captured outside}{captured within}*\frac{traps inside}{traps outside}*{month}^{-1}$.

We want the proportion of fish leaving per year. Say $p_{m}$ is the proportion of fish staying per month, then $spillover=\frac{1-p_{m}}{p_{m}}\Rightarrow p_{m}=\frac{1}{1+spillover}\Rightarrow for each year p_{y}=\left( p_{m} \right)^{12}=\left( \frac{1}{1+spillover} \right)^{12}$. The values of spillover given in the paper are 0.003, 0.01, 0.04, 0.07, 0.25 (Table 4) which translate to

$p_{y}=0.965,0.887, 0.625, 0.444, 0.07$, respectively. We can find the exponential parameter and compare to the spillover parameter in our paper:

$\dot{P}=-mP\Rightarrow P\left( t \right)=P_{0}e^{-mt}\underset{t=1 year}{\underbrace{=}}p_{y}P_{0}\Rightarrow m=-\ln\left( p_{y} \right)$. We get $m=0.036, 0.12, 0.47,0.812, 2.66$, respectively.

References

Kaunda-Arara B, Rose GA (2004) Out-migration of tagged fishes from marine reef National Parks to fisheries in coastal Kenya. Environmental Biology of Fishes 70:363-372
