# Supplementary material for: Potential contribution of fish restocking to the recovery of deteriorated coral reefs: an alternative restoration method?
Source: PeerJ. 2016 Feb 29;4:e1732. doi: 10.7717/peerj.1732 (PMC4782690; doi:10.7717/peerj.1732)

Figure S2.1: the dynamics are simulated with the same parameter values as in figure 1 in the main text, with the exception of the discount rate, which is taken as $d^{'}=0.01$.


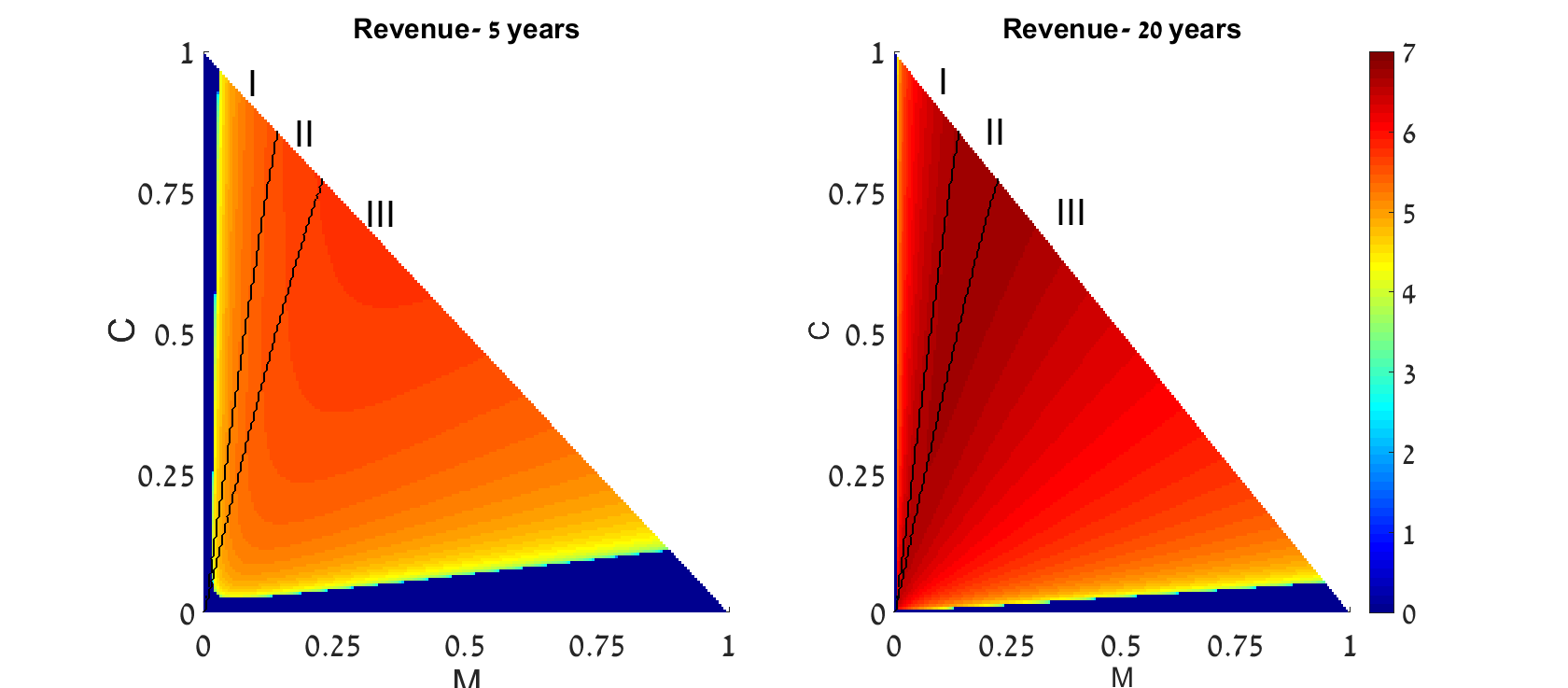


Figure S2.2: the dynamics are simulated with the same parameter values as in figure 1 in the main text, with the exception of the discount rate, which is taken as $d^{'}=0.07$.


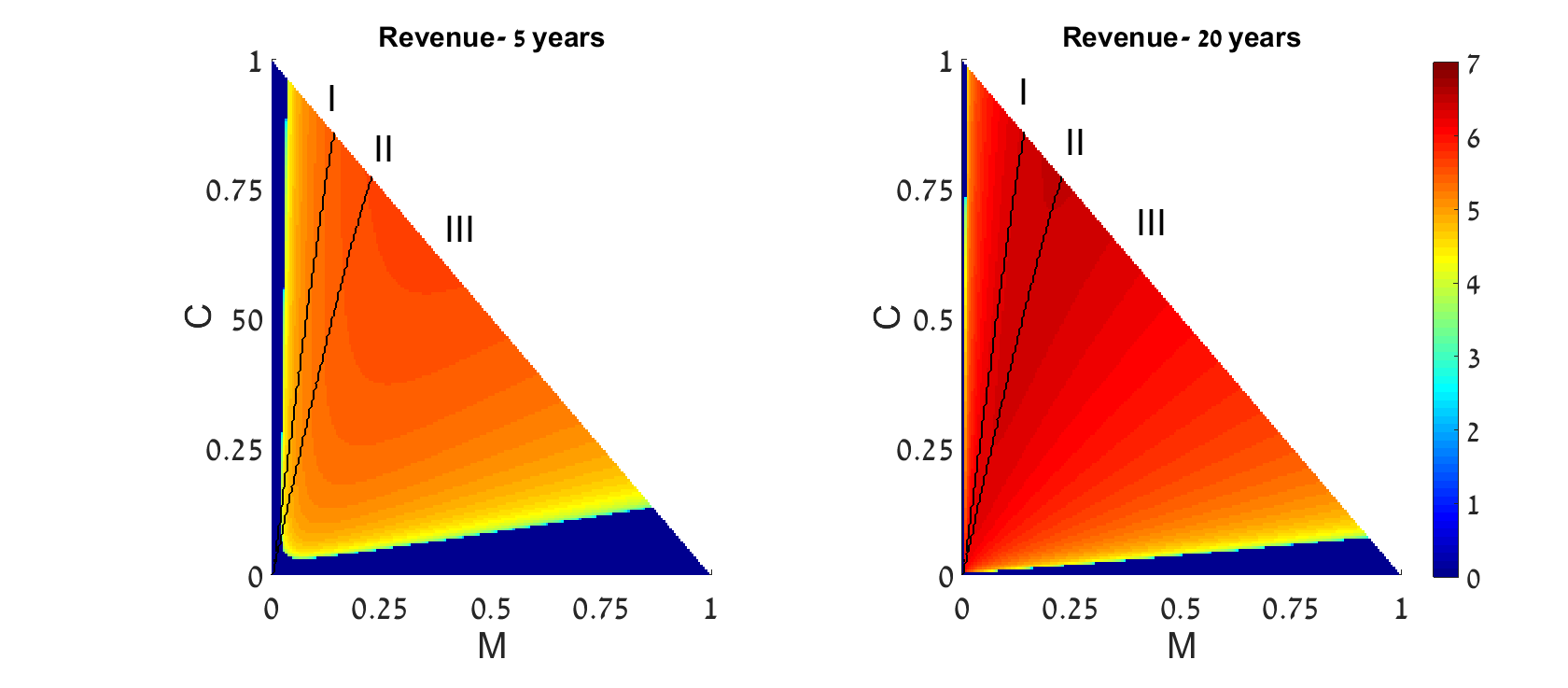

Supplement: Text S2 [file peerj-04-1732-s002.docx]
